# Alpha-Linolenic Acid Modulates T Cell Incorporation in a 3D Tissue-Engineered Psoriatic Skin Model

**DOI:** 10.3390/cells11091513

**Published:** 2022-04-30

**Authors:** Sophie Morin, Mélissa Simard, Geneviève Rioux, Pierre Julien, Roxane Pouliot

**Affiliations:** 1 Centre de Recherche en Organogénèse Expérimentale de l’Université Laval/LOEX, Axe Médecine Régénératrice, Centre de Recherche du CHU de Québec-Université Laval, 1401 18e Rue, Québec City, QC G1J 2Z4, Canada; sophie.morin.7@ulaval.ca (S.M.); melissa.simard.06@gmail.com (M.S.); genevieve.rioux.9@ulaval.ca (G.R.); 2 Faculté de Pharmacie, Université Laval, Québec City, QC G1V 0A6, Canada; 3 Centre de Recherche du CHU de Québec-Université Laval, Axe Endocrinologie et Néphrologie, Université Laval, Québec City, QC G1V 4G2, Canada; pierre.julien@crchudequebec.ulaval.ca; 4 Département de Médecine, Faculté de Médecine, Université Laval, Québec City, QC G1V 0A6, Canada

**Keywords:** psoriasis, tissue engineering, T cells, n-3 PUFAs

## Abstract

Psoriasis is an autoimmune skin disease with an increased number of leukocytes infiltrating the dermal and epidermal compartments compared with normal skin. N-3 polyunsaturated fatty acids (n-3 PUFAs) are frequently used in the clinic in order to attenuate the symptoms of psoriasis. For psoriatic patients, a supplementation of the diet with alpha-linolenic acid (ALA) reduces the activation of T cell signaling pathways, leading to a significant reduction in inflammatory cytokine secretion. However, the precise mechanism of action of n-3 PUFAs in psoriasis is still not understood. In the present study, we elucidated the bioaction of ALA on the adaptive immune component of psoriasis by using a psoriatic skin model produced with the addition of activated T cells. Healthy and psoriatic skin substitutes were produced according to the self-assembly method, using culture media supplemented with 10 μM of ALA. T cells were isolated from blood samples using a negative selection isolation method. ALA supplementation regulated the hyperproliferation and abnormal cell differentiation of psoriatic keratinocytes stimulated by T cells. Additionally, the exogenous ALA was correctly incorporated into the phospholipids of keratinocytes, which resulted in increased levels of ALA, eicosapentaenoic acid (EPA) and n-3 docosapentaenoic acid (n-3 DPA). The infiltration of T cells into the epidermis was reduced when ALA was added to the culture medium, and significant decreases in the levels of inflammatory cytokines and chemokines such as CXCL1, interleukin-6 (IL-6) and interleukin-8 (IL-8) were consequently measured in psoriatic substitutes supplemented with this n-3 PUFA. Altogether, our results showed that in this psoriatic skin model enriched with T cells, ALA exerted its beneficial effect by decreasing the quantities of inflammatory mediators released by T cells.

## 1. Introduction

Psoriasis is a very common chronic inflammatory skin disease affecting about 3% of the population worldwide [1]. The main pathological alterations appear in the form of symmetrical inflammatory lesions. Multiple erythematous scaly and well-defined plaques develop on the elbows, knees and scalp, but the disease can spread throughout the entire body. Psoriatic plaques can lead to even more harmful consequences, including a noticeable decrease in the patient’s quality of life [2,3]. Psoriasis presents histopathological changes in almost all types of skin cells, but psoriatic plaques are mainly characterized by the hyperproliferation and abnormal cell differentiation of keratinocytes, leading to epidermal thickening. The differentiation program of keratinocytes is extensively altered in psoriasis. In this regard, the expression of epidermal differentiation proteins, such as filaggrin, loricrin, involucrin and transglutaminase 1 (TGM1), is deregulated in psoriatic skin [4]. In addition, the pathology is distinguished by an inappropriate immune activation, ultimately leading to extensive leukocyte infiltration into the dermis and epidermis, mainly by T cells, dendritic cells and monocytes. The interactions between immune cells and keratinocytes allow the development and maintenance of psoriatic plaques [4,5,6]. Indeed, psoriasis is a well-known T cell-driven disease, but the chemokines produced by keratinocytes are essential for continuing the inward migration of leukocytes into the epidermis, considering that the diverse mediators produced by epidermal keratinocytes directly affect T cell functions [7,8]. In addition, the immunological alterations in psoriasis lead to the production of high amounts of various cytokines, including tumor necrosis factor alpha (TNF-α), which is produced by keratinocytes and T cells, as well as interferon-gamma (IFN-γ), interleukin-17A (IL-17A) and interleukin-22 (IL-22), which are produced mainly by T cells. These latter promote the release of other inflammatory cytokines, culminating in the activation of naive T cells that will proliferate and migrate to inflammatory sites. In psoriatic skin, cytokines and chemokines act as key molecular coordinators that dictate the behavior of psoriatic cells [8,9]. Following their binding to their corresponding chemokine receptors, chemokine ligands and cytokines migrate into inflamed psoriatic skin along chemokine gradients, contributing to local and systemic inflammation [10]. In the epidermis of psoriatic skin, TNF-α stimulates keratinocytes to produce CXCL1, CXCL2 and IL-8, which activate T cells and help to maintain the proliferation of Th17 cells [7]. Supra-basal keratinocytes express the chemokine receptor CXCR2 and the CXCL1/CXCR2 axis triggers the activation of the mitogen-activated protein (MAP) kinase signaling proteins, which play a pivotal role in amplifying the psoriatic inflammatory response [10].

Moreover, the inflammatory component of psoriasis is reflected in a noteworthy deregulation of the skin lipid mediator profile, first and foremost in the inflammatory cascade of arachidonic acid (AA), especially with increased levels of leukotriene B_4_ (LTB_4_) and prostaglandin E2 (PGE_2_) in psoriatic lesions [11,12]. PGE_2_ promotes the development of psoriasis through the regulation of the IL-23/IL-17 pathway in T cells [13]. In the last decade, several experimental and clinical studies have suggested that n-3 polyunsaturated fatty acids (n-3 PUFAs) have many beneficial effects on diverse autoimmune disorders. In fact, a high n-3 PUFA intake was shown to ameliorate psoriatic symptoms, essentially by diminishing the epidermal thickness and scaliness of psoriatic plaques [14,15,16]. N-3 PUFAs compete with n-6 PUFAs in the metabolic pathway for their incorporation into cell membranes, leading the profile of lipid metabolites towards an anti-inflammatory one [11]. Additionally, n-3 PUFAs can reduce inflammation by interfering with T-cell signaling pathways [17,18]. In fact, in 1996, Jeffery et al. were among the first to demonstrate that the supplementation of rats’ diet with 200 g/kg of n-3 PUFAs over a 6-week period results in a marked diminution of spleen T cell proliferation [19,20]. Over the last decade, different studies have reported that the oral administration of EPA and docosahexaenoic acid (DHA) in humans could diminish the number of activated T cells among the peripheral blood mononuclear cells (PBMCs) of healthy subjects [21,22]. A diet rich in n-3 PUFAs is consequently able to reduce the production of inflammatory cytokines in PBMCs, such as IL-1, TNF-α and IL-6 and can downregulate the expression of several adhesion molecules, particularly MCP-1, which is normally stimulated by TNF-α [23,24,25]. However, alpha-linolenic acid (ALA), the precursor of EPA and DHA, has rarely been evaluated for these effects and the exact mechanism of action by which n-3 PUFAs regulate human T cells in the pathology of psoriasis is still not well detailed in the literature.

To this day, it is well recognized that n-3 PUFAs can decrease the hyperplasia of psoriatic epidermal cells [26]. In our latest study, we have demonstrated that the supplementation of the culture media with ALA reduces proliferation and restores the cell differentiation of psoriatic keratinocytes in our three-dimensional in vitro human psoriatic skin model [27]. However, the exact bioaction of ALA on the immune component of our psoriatic skin model, which is created by the addition of T cells, has still not been characterized. Since the inflammation triggered by the interaction between T cells and psoriatic keratinocytes is a hallmark of the pathogenesis of psoriasis, we decided to further study the supplementation of the culture media with ALA in our reconstructed psoriatic skin model enriched with T cells. Despite some limitations of this model, among others the use of activated healthy T cells, it appears that psoriatic keratinocytes allow the polarization of the T cells added to the model towards an inflammatory profile [28]. Therefore, the aim of this study was to investigate the biological activity of ALA that affects the cell differentiation and proliferation of psoriatic keratinocytes specifically cultivated with activated T cells. The current study was also intended to evaluate the biological action of ALA on psoriatic characteristics that are regulated by T cells, namely the secretion of inflammatory cytokines and the migration of T cells, by using our immunocompetent psoriatic skin model [28].

## 2. Materials and Methods

### 2.1. Biopsies and Donors

Three healthy patients aged 38, 39 and 46 years old were recruited. Skin cells were extracted from healthy donors, who were all Caucasian females, after breast reduction surgeries. Psoriatic skin substitutes were produced using epithelial cells obtained from psoriatic biopsies from Caucasian donors (donors aged 36 (female), 49 (male) and 64 years old (female)). Psoriatic donors all had plaque psoriasis. All biopsies were obtained in agreement with the Declaration of Helsinki and performed under the guidelines of the Research Ethics Committee of the Centre Hospitalier Universitaire (CHU) de Québec. All donors were given adequate information for providing written consent. Fibroblasts were isolated from the extract using thermolysin and collagenase. As for keratinocytes, they were isolated by using thermolysin and trypsin [29].

### 2.2. Cell Culture Condition

Dermal cells (fibroblasts) were cultured in Dulbecco-Vogt modification Eagle’s medium (DMEM) supplemented with 10% fetal bovine serum (FBessence, Seradigm, Mississauga, ON, Canada), 100 Ul/mL penicillin G (Sigma, Oakville, ON Canada), 25 µL/mL gentamicin (Schering, Pointe-Claire, QC, Canada) and 50 μg/mL of ascorbic acid (Sigma-Aldrich, St-louis, MO, USA). Epidermal cells (keratinocytes) were cultured in DMEM with Ham’s F12 (3:1) supplemented with 5% Fetal Clone II serum (HyClone, ThermoFisher Scientific, Ottawa, ON, Canada), 100 UI/mL penicillin G (Sigma, Oakville, ON, Canada), 25 µg/mL gentamicin (Schering, Pointe-Claire, QC, Canada), 5 µg/mL insulin (Sigma, Oakville, ON, Canada), 0.4 µg/mL hydrocortisone (Calbiochem, EMD, Biosciences, Gibbstown, NJ, USA), 10 ng/mL human epidermal growth factor (EGF; Austral Biological, San Ramon, CA, USA) and 10^−^^10^ M cholera toxin (MP Biomedicals, Montreal, QC, Canada). For n-3 PUFA supplementation, ALA (Sigma-Aldrich, St-Louis, MO, USA) was dissolved in 99% ethanol (EtOH) (Greenfield, Global, Brampton, ON, Canada). Then, the ALA-EtOH solution was added to the culture media at a concentration of 10 µM, which was previously determined using dose–response assays [30]. Healthy and psoriatic skin substitutes enriched in T cells or not were produced with media supplemented with either ALA (PS^+T+ALA^) or with an equivalent volume of evaporated EtOH (HS, PS and PS^+T^).

### 2.3. Isolation and Activation of T Cells

T cells were isolated from human whole blood by immunomagnetic negative selection of T cells. The EasySep™ Direct Human T Cell Isolation Kit (StemCell Technologies, Vancouver, BC, Canada) was used in order to isolate T cells from whole blood obtained from healthy donors (blood donors were not matched with the psoriatic cell donors). Three healthy donors were recruited for blood samples. All blood samples were obtained in agreement with the Declaration of Helsinki and under the guidelines of the Research Ethics Committee of the Centre Hospitalier Universitaire (CHU) de Québec. The procedure was carried out as indicated by the manufacturer. This process was performed at room temperature and in a sterile environment. Once isolated, the T cells were activated by a stimulation method using phorbol 12-myristate 13-acetate (PMA) and ionomycin. The T cells were then cultured in DMEM media supplemented with 25 ng/mL of PMA (Sigma-Aldrich, St-Louis, MO, USA) and 1 µg/mL of ionomycin (Sigma-Aldrich, St-Louis, MO, USA) for 4 h at 37 °C. After 4 h of incubation, the T cells were washed and seeded on fibroblast sheets at a concentration of 0.5 × 10^6^ cells/substitute [28].

### 2.4. Skin Substitutes Production Using the Self-Assembly Method

The self-assembly method was used in order to produce healthy and psoriatic skin substitutes as previously described [28,31]. The protocol is as follows: Human fibroblasts (1.2 × 10^5^) were seeded into 6-well plates and cultured over a period of 3 weeks at 37 °C, 8% CO_2_, so that they were able to form their own extracellular matrix. T cells (0.5 × 10^6^ cells), isolated and activated as explained previously, were seeded on one fibroblast sheet out of two and the cell assembly was cultured for 7 more days in the presence of 30 U/mL recombinant human IL-2 (R & D Systems, Minneapolis, MN, USA). Meanwhile, keratinocytes (1.2 × 10^6^ cells) were seeded on the other fibroblast sheet of the two (the fibroblast sheet without T cells). Keratinocytes were cultured over 7 days (the same week as the fibroblast and T cell assembly) and the culture media were changed every day. After that, the sheet containing the keratinocytes was superimposed onto the fibroblast sheet containing the T cells. The skin equivalent was then raised to the air–liquid interface, allowing the keratinocytes to be in contact with the air. The air–liquid interface culture was maintained for a total period of 3 weeks using DMEM with Ham’s F12 (3:1) supplemented with 5% Fetal Clone II serum (HyClone, ThermoFisher Scientific, Ottawa, ON, Canada), 100 Ul/mL penicillin G (Sigma, Oakville, ON, Canada), 25 μg/mL gentamicin (Schering, Pointe-Claire, QC, Canada), 5 μg/mL insulin (Sigma, Oakville, ON, Canada), 0.4 μg/mL hydrocortisone (Calbiochem, EMD, Biosciences, Gibbstown, NJ, USA) and 10^−^^10^ M cholera toxin (MP Biomedicals, Montreal, QC, Canada). The keratinocytes were then able to differentiate completely [28].

### 2.5. Histology and Immunofluorescence Analyses

For histology, fragments of each skin substitute were fixed in a formol solution (ThermoFisher Scientific, Waltham, MA, USA) and embedded in paraffin. After that, hematoxylin and eosin staining were performed on 6 µm-thick sections. The thickness of the living epidermis of each skin substitute was measured with ImageJ software version 1.52a (National Institutes of Health, Bethesda, MD, USA, http://imagej.nih.gov/ij) (accessed on 5 January 2020). The thickness of the living epidermis was measured in duplicate for three different cell populations and 10 measurements per condition were made (N = 3, *n* = 2, 10 measurements for each skin substitute). For immunofluorescence staining, samples were submerged in Tissue-Tek O.C.T compound (Sakura Finetek, Torrance, CA, USA) and frozen in liquid nitrogen. Indirect immunofluorescence was carried out on 6 µm cryosections after a 10 min incubation in acetone at −20 °C. The antibodies were prepared using phosphate-buffered saline (PBS) containing 1% bovine serum albumin (BSA). The samples were incubated for 45 min at room temperature with the primary antibodies in a dark chamber and then for 30 min with the secondary antibodies in the same conditions. The complete list of antibodies used is detailed in the supplementary material (see Supplementary Table S1). Cell nuclei were counterstained with a 4′-6-diamidino-2-phenylindole (DAPI) reagent that was added directly to the mounting medium. Negative controls of each secondary antibody (Anti-mouse Alexa 488, Anti-rabbit Alexa 488 and Streptavidin Alexa 594) were performed on sections of normal human skin and are shown in Figure S2. All slides analyzed were observed with a Zeiss Axio Imager (Carl Zeiss Canada Ltd., Toronto, ON, Canada) with controlled parameters, including exposure times.

### 2.6. Western Blot Analysis

After separation of the dermis from the epidermis using scalpels and forceps, both skin compartments were ground separately using a cryogenic grinder (Cryomill MM400; Retsch^®^, Newtown, PA, USA). The total proteins of each ground sample (dermis and epidermis) were extracted with RIPA buffer containing a protease inhibitor cocktail (Ocomplete, Roche, Diagnostics GmbH, Germany). Ten percent polyacrylamide gels were prepared using 20 μg of total proteins and were then resolved by SDS-PAGE. The gels were transferred onto PVDF membranes (Bio-Rad, Hercules, CA, USA) overnight. Each membrane was blocked for 1 h in tris-buffered saline (TBS) containing 5% non-fat milk and 0.05% Tween 10. Then, the membranes were incubated with primary antibodies for 1 h at room temperature, followed by a 1 h incubation at room temperature with HRP-labeled secondary antibodies (see Supplementary Table S1). The Amersham ECL Western Blotting Detection Reagent (GE Healthcare, Little Chalfont, UK) was used to detect protein expression on each membrane. A Fusion Fx7 imager (MIB Lab Equipment, Kirkland, QC, Canada) was used to view the different bands from the blots and each band was quantified using ImageJ software version 1.52a (National Institutes of Health, Bethesda, MD, USA, http://imagej.nih.gov/ij) (accessed on 5 January 2020). The complete list of antibodies used is detailed in the supplementary material (see Supplementary Table S1).

### 2.7. Gas Chromatography (GC-FID) Analysis

Epidermal samples were analyzed using a gas chromatography with flame ionization detector (GC-FID) as described elsewhere [27,32,33]. The epidermis was separated from the dermis with scalpels and then incubated in water for 1 min. The solution used to extract the lipids from the epidermis was a mixture of chloroform and methanol (2:1 *v*/*v*) (modified from the Folch method). First, phospholipids were separated by thin layer chromatography and then the fatty acids were methylated. Gas chromatography was performed using a HP5890 gas chromatograph (Hewlett-Packard, Toronto, ON, Canada) with a HP-88 capillary column (Agilent Technologies, Santa Clara, CA, USA) coupled with a flame ionization detector. 

### 2.8. Cytokine Array

The Human Cytokine Array (R & D Systems, Minneapolis, MN, USA) was used following the manufacturer’s protocol. A total of 36 cytokines were analyzed. The supernatants of cell culture media were collected on the first day of air–liquid culture and stored at −80 °C. A total of 600 μL of cell culture supernatant was used to detect the cytokine on each array membrane. The cytokines were detected with a Fusion Fx7 over a period of 15 min (MIB Lab Equipment, Kirkland, QC, Canada). The different cytokines detected were quantified using ImageJ software version 1.52a (National Institutes of Health, Bethesda, MD, USA, http://imagej.nih.gov/ij (accessed on 30 January 2020)).

### 2.9. ELISA Assays

The ELISAs were performed using 7-day air–liquid culture supernatants. The levels of TNF-α and IFN-γ were assayed using a TNF-α Competitive ELISA Kit (ThermoFisher Scientific, Waltham, MA, USA) and an IFN-γ Competitive ELISA kit (ThermoFisher Scientific, Waltham, MA, USA), respectively. A volume of 150 µL of supernatant was used in the TNF-α assay and a volume of 50 µL of supernatant in the IFN-γ assay.

### 2.10. Statistical Analysis

Statistical analyses were performed using Prism8 software (GraphPad Software, La Jolla, CA, USA). Data were expressed as means ± standard deviations (SD), while statistical analyses were carried out using analyses of variance (ANOVAs) followed by Tukey’s post hoc test. The *p*-values < 0.05 were considered as statistically significant.

## 3. Results

### 3.1. ALA Regulates the Hyperproliferation of Psoriatic Keratinocytes in the Presence of T Cells

**Figure 1:**
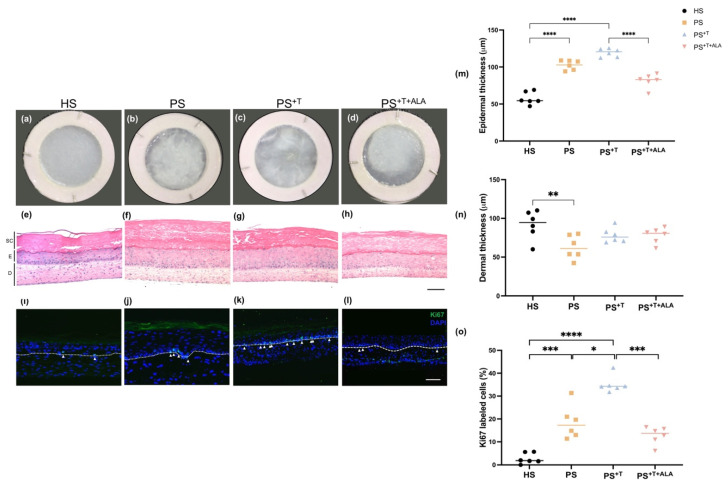
Histological aspects of HS, PS, PS^+T^ and PS^+T+ALA^. (**a**–**d**) Macroscopic representations of healthy and psoriatic skin substitutes; (**e**–**h**) Hematoxylin and eosin (H & E) staining of healthy and psoriatic skin substitutes. Scale bar represents 100 μm. (SC, stratum corneum; E, epidermis; D, dermis); (**i**–**l**) Immunofluorescence staining of Ki67 showing proliferation in healthy and psoriatic skin substitutes. Scale bar: 100 µm. Arrows show the labeled cells. The dotted lines show the dermo-epidermal junction; (**m**) Quantification of the living epidermal thickness of the skin substitutes; (**n**) Quantification of the dermal thickness of the skin substitutes; (**o**) Ratio of Ki67 positive cells to the number of total keratinocytes in the basal layer. The values are presented as mean ± SD (N = 3 donors, *n* = 2 skin substitutes per donor). Statistical significance was determined using two-way ANOVA followed by Tukey’s post hoc test. Significant differences are indicated by asterisks (* *p* < 0.05; ** *p* < 0.01; *** *p* < 0.001, **** *p* < 0.0001). Abbreviations: ALA; alpha-linolenic acid; HS: healthy substitutes; T: T cells; PS: psoriatic substitutes; PS^+T^: psoriatic substitutes produced with T cells; PS^+T+ALA^: psoriatic substitutes produced with T cells and supplemented with ALA.

Macroscopic analyses of skin substitutes showed that psoriatic substitutes (PS) and psoriatic substitutes produced with T cells (PS^+T^) had a rougher and less homogeneous surface compared with healthy substitutes (HS) (Figure 1a–c). On the other hand, psoriatic substitutes produced with T cells and supplemented with ALA (PS^+T+ALA^) were closer to a healthy appearance, with a smoother and more opaque surface (Figure 1d). According to the histological aspects of the skin substitutes, PS and PS^+T^ had a significantly thicker epidermis than HS, confirming the hyperproliferation of psoriatic keratinocytes in our model (Figure 1e–g,m). Interestingly, PS^+T^ had a thicker living epidermis compared with PS, though not significant, suggesting the involvement of T cells in the excessive proliferation process of psoriatic keratinocytes. The histology of PS^+T^ therefore emphasizes the importance of the interactions between keratinocytes and T cells. The epidermis of PS^+T+ALA^ was not as thick as its counterpart PS^+T^, suggesting decreased acanthosis following ALA supplementation (Figure 1g,h,m). A decrease in the dermal thickness was measured in PS compared with HS (Figure 1e,f,n). Moreover, PS and PS^+T^ had more keratinocytes labeled with the proliferation marker Ki67 in their basal membrane than HS, confirming the hyperproliferation of psoriatic keratinocytes in the model (Figure 1i–k,o). More basal keratinocytes were labeled with Ki67 in PS^+T^ than in PS (Figure 1i–k,o). We cannot exclude that some T cells might have migrated to the basal layer of the epidermis and thus have contributed to the higher number of Ki67 positive cells in PS^+T^, but the fact remains that the presence of ALA visibly decreased basal cell proliferation. Indeed, the ability of ALA to decrease the hyperproliferation of psoriatic keratinocytes was maintained even in the presence of activated T cells, since a decrease in the number of Ki67-labeled cells was observed in the basal membrane of PS^+T+ALA^ compared with PS^+T^ (Figure 1l,o). Finally, keratin 17 (K17) is an intermediate protein found in healthy basal keratinocytes and its expression spreads aberrantly into the supra-basal psoriatic keratinocytes [34]. K17 was highly expressed in PS and PS^+T^ when compared with HS. Moreover, ALA supplementation restored the differentiation program of psoriatic keratinocytes, since the expression of K17 was significantly downregulated in PS^+T+ALA^ compared with PS^+T^ and PS (Figure S1).

### 3.2. Restoration of Epidermal Cell Differentiation Proteins following ALA Supplementation in Psoriatic Substitutes

**Figure 2:**
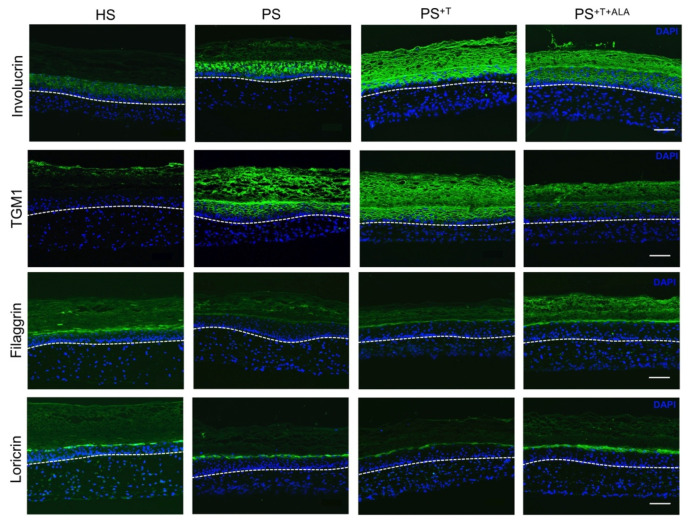
Immunofluorescence staining of cell differentiation proteins in skin substitutes. Indirect immunofluorescence staining was carried out on HS, PS, PS^+T^ and PS^+T+ALA^. Nuclei were counterstained with DAPI reagent (blue). Dashed white lines represent the basement membrane. Scale bar: 100 µm. Abbreviations: ALA: alpha-linolenic acid; HS: healthy substitutes; T: T cells; PS: psoriatic substitutes; PS^+T^: psoriatic substitutes produced with T cells; PS^+T+ALA^: psoriatic substitutes produced with T cells and supplemented with ALA; TGM1: transglutaminase 1.

In order to evaluate the effects of ALA on the cell differentiation program of the psoriatic epidermis, we investigated the expression of cell differentiation proteins in all skin substitutes (Figure 2). Involucrin and TGM1 expressions were up-regulated in PS and PS^+T^ when compared with HS (Figure 2), as observed in native psoriatic skin, since these are markers of early differentiation [35]. Moreover, the expression of both proteins was increased in PS^+T^ when compared with PS, suggesting that T cells play a role in the disturbed cell differentiation found in psoriasis [36]. On the other hand, the addition of ALA to the culture media reduced the expression of involucrin and TGM1 in PS^+T+ALA^ to a level closer to that of HS. Conversely, loricrin and filaggrin are markers of late differentiation in the epidermis, which explains the diminution of their expression in PS and PS^+T^. In fact, filaggrin was almost completely absent in PS and PS^+T^. The addition of exogenous ALA increased the expression of both filaggrin and loricrin in PS^+T+ALA^, thus normalizing their synthesis in psoriatic substitutes.

### 3.3. Effects of ALA Supplementation on the Epidermal n-3 and n-6 Phospholipids of the Skin Substitutes

**Figure 3:**
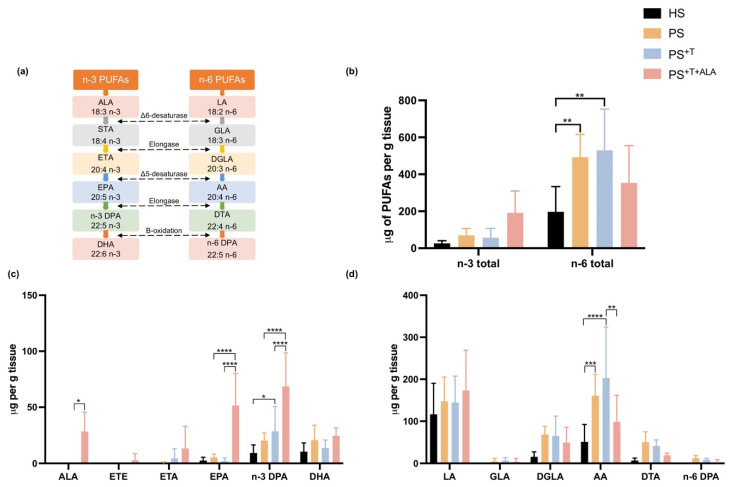
PUFA incorporation into the phospholipid fraction of the epidermis of skin substitutes. Effects of supplementation with ALA and T on the levels of n-3 and n-6 PUFAs in the epidermal phospholipids of HS, PS, PS^+T^ and PS^+T+ALA^. (**a**) Metabolic pathways of n-3 and n-6 PUFAs; (**b**) Total levels of n-3 and n-6 PUFAs in skin substitutes; (**c**) n-3 PUFA levels in the epidermal phospholipids of skin substitutes; (**d**) n-6 PUFA levels in the epidermal phospholipids of skin substitutes. PUFAs were quantified by gas chromatography and results are presented as μg per g of tissue. (N = 3 donors, *n* = 2 skin substitutes per donor). Statistical significance was determined using two-way ANOVA followed by Tukey’s post hoc test. Significant differences are indicated by asterisks (* *p* < 0.05; ** *p* < 0.01; *** *p* < 0.001, **** *p* < 0.0001). Abbreviations: AA: arachidonic acid; ALA: alpha-linolenic acid; DGLA: dihomo-γ-linolenic acid; DHA: docosahexaenoic acid; DPA: docosapentaenoic acid; DTA: docosatetraenoic acid; EPA: eicosapentaenoic acid; ETA: eicosatetraenoic acid; ETE: eicosatrienoic acid; GLA: γ-linolenic acid; HS: healthy substitutes; LA: linoleic acid; T: T cells; n-3: omega-3; n-6: omega-6; PUFAs: polyunsaturated fatty acids; PS: psoriatic substitutes; PS^+T^: psoriatic substitutes produced with T cells; PS^+T+ALA^: psoriatic substitutes produced with T cells and supplemented with ALA.

The two precursors of n-3 and n-6 PUFAs, ALA and linoleic acid (LA), use parallel metabolic pathways and compete for the same enzymes that desaturate and elongate them into long-chain PUFAs (Figure 3a). A gas chromatograph with a flame ionization detector (GC-FID) was used to study the incorporation of ALA into the phospholipid fraction of the epidermis of the skin substitutes (Figure 3b–d). Higher levels of total n-3 PUFAs were quantified in the epidermal phospholipid fraction after ALA supplementation in PS^+T+ALA^ (Figure 3b). In fact, the amounts of ALA, EPA and n-3 docosapentaenoic acid (n-3 DPA) were significantly higher in the phospholipid fraction of the epidermis of PS^+T+ALA^ compared with its counterpart PS^+T^ (+25-fold, 12.6-fold and 2.4-fold, respectively) (Figure 3c). Regarding the metabolism of n-6 PUFAs, higher amounts of arachidonic acid (AA) were quantified in PS and PS^+T^ compared with HS, which agrees with studies showing that AA and its derived metabolites are found in increased quantities in psoriasis (Figure 3d) [12,37]. Although not statistically significant, the amount of AA tended to be higher in PS^+T^, indicating an inflammatory environment engendered by the addition of T cells. The supplementation of the culture media with ALA (PS^+T +ALA^) significantly reduced the over-production of AA measured in PS^+T^ (Figure 3d). Thus, ALA competed with the n-6 metabolic pathway in the psoriatic substitutes produced with T cells. Levels of fatty acids in phospholipids of the skin substitutes after ALA supplementation are detailed in Table S2.

### 3.4. Impact of ALA on the Addition of T Cells to Psoriatic Skin Substitutes

**Figure 4:**
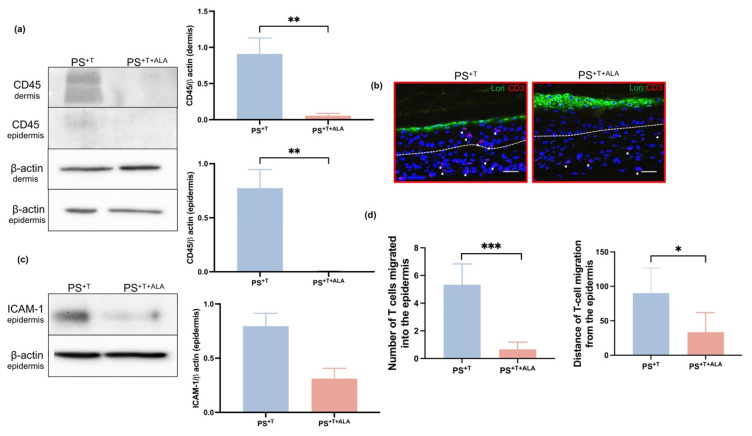
Expression of T cell markers in psoriatic skin substitutes. (**a**) Twenty micrograms of total protein from skin substitutes were analyzed by immunoblot for the presence of CD45 in the epidermis and the dermis of PS^+T^ and PS^+T+ALA^. β-actin was used to control equal loading. One representative immunoblot is shown per protein (N = 3 donors per condition; *n* = 2 skin substitutes per donor); (**b**) Indirect immunofluorescence staining was conducted on PS^+T^ and PS^+T+ALA^. The expression of CD3 is shown in red, while the expression of loricrin is shown in green and nuclei were counterstained with DAPI reagent (blue). The arrows show the CD3-labeled cells and the dashed white lines represent the basement membrane. Scale bars: 100 µm; (**c**) Twenty micrograms of total protein from skin substitutes were analyzed by immunoblot for the presence of ICAM-1 in the epidermis of PS^+T^ and PS^+T+ALA^. β-actin was used to control equal loading. One representative immunoblot is shown per protein (N = 3 donors per condition; *n* = 2 skin substitutes per donor); (**d**) Number of T cells having migrated into the epidermis as well as their distance of migration in the epidermis (relative to the basal membrane) in PS^+T^ and PS^+T+ALA^. Student’s t-tests were performed for statistical analyses. Significant differences are indicated by asterisks (* *p* < 0.05; ** *p* < 0.01; *** *p* < 0.001). Abbreviations: ALA: alpha-linolenic acid; ICAM-1: Intercellular adhesion molecule; Lori: Loricrin; T: T cells; PS^+T^: psoriatic substitutes produced with T cells; PS^+T+ALA^: psoriatic substitutes produced with T cells and supplemented with ALA.

In order to partially quantify the presence of T cells in the psoriatic skin model, the levels of CD45 in the dermis and epidermis were analyzed by Western-blot, since CD45 is among the most abundant proteins in the T cell plasma membrane [12,38]. In fact, T cells require CD45 to detect and respond to different antigens [39]. In the dermis, the amount of CD45 was increased in PS^+T^ compared with PS^+T+ALA^, showing that ALA reduced the expression of CD45 on the surface of T cells, which suggests that ALA potentially decreased the number of T cells in the psoriatic skin model (Figure 4a and Figure S3). The expression of CD45 likely belongs to T cells since they were the only leukocytes added to the model. In the epidermis, CD45 was poorly detected in PS^+T^ and no expression was detected in PS^+T+ALA^, suggesting that the presence of ALA in the culture medium decreased the movement of T cells in the psoriatic model (Figure 4a and Figure S3). To validate the results obtained by Western-blot, we performed more precise labeling of T cells by immunofluorescence staining for the T cell marker CD3 (Figure 4b). After 21 days at the air–liquid interface, T cells were still present in the dermis of the skin substitutes, as they were detected in PS^+T^ and PS^+T+ALA^. Thus, T cells were properly incorporated into the dermal compartment of both PS^+T^ and PS^+T+ALA^. In addition, T cells tended to migrate towards the epidermis in PS^+T^, since many CD3-labeled cells were located past the dermo-epidermal junction (Figure 4b). On the other hand, CD3-labeled cells were mainly present in the deeper layer of the dermis in PS^+T+ALA^ and no T cells were found in the epidermal compartment, suggesting that the presence of ALA slowed down the migration of T cells towards the epidermis. Since a diminution in T cell migration was observed following ALA supplementation, we studied the expression of a leukocyte–endothelial cell interaction marker, the intracellular adhesion molecule 1 (ICAM-1) [40]. In accordance with the CD3-labeling results, the expression of ICAM-1 was higher in PS^+T^ when compared with PS^+T+ALA^. A diminution of ICAM-1 expression was observed in PS^+T+ALA^, explaining at least in part the reduction in T cell migration following ALA supplementation. Finally, the infiltration of T cells into the epidermis was analyzed in PS^+T^ and PS^+T+ALA^ by counting the number of T cells present in the epidermis of the skin substitutes as well as by measuring their distance of migration from the basal membrane of the epidermis. A reduction in the number of T cells and in migratory distance was quantified in PS^+T+ALA^ compared with PS^+T^ (Figure 4d).

### 3.5. Impact of ALA Supplementation on Inflammatory Cytokine Secretion by T Cells and Downstream Signaling in Psoriatic Substitutes

**Figure 5:**
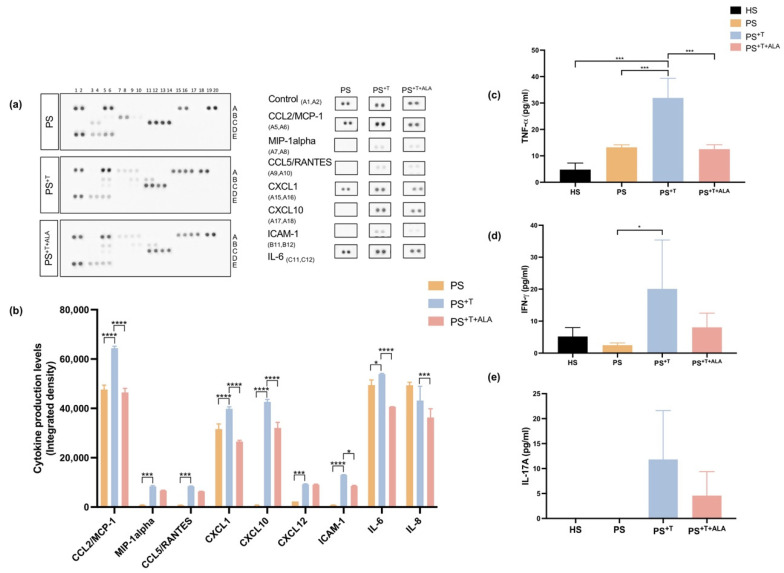
Levels of inflammatory cytokines in PS, PS^+T^ and PS^+T+ALA^. (**a**) Culture supernatants from PS, PS^+T^ and PS^+T+ALA^ were used to detect secreted cytokines using the Human Cytokine Array kit from R&D Systems. The duplicate spots correspond to the cytokines whose synthesis was the most altered following ALA supplementation; (**b**) Densitometric analysis of the dot blot duplicates from panel (**a**); (**c**) Tumor necrosis factor-α (TNF-α) levels in the culture medium of skin substitutes (N = 3 donors per condition, *n* = 2 culture supernatants per donor); (**d**) Interferon-gamma (IFN- γ) levels in the culture medium of skin substitutes (N = 3 donors per condition, *n* = 2 culture supernatants per donor); (**e**) IL-17A levels in the culture medium of skin substitutes (N = 2 donors per condition, *n* = 1 culture supernatant per donor). Statistical significance was determined using two-way ANOVA followed by Tukey’s post hoc test. Significant differences are indicated by asterisks (* *p* < 0.05, *** *p* < 0.001, **** *p* < 0.0001). Abbreviations: ALA: alpha-linolenic acid; CCL: chemokine ligand; CXCL: chemokine C-X-C motif ligand; HS: healthy substitutes; ICAM: intercellular adhesion molecule; IL: interleukin; T: T cells; PS: psoriatic substitutes; PS^+T^: psoriatic substitutes produced with T cells; PS^+T+ALA^: psoriatic substitutes produced with T cells and supplemented with ALA.

Recently, our group has shown that psoriatic substitutes present a cytokine profile which corresponds to that of native psoriatic skin [28]. In the present study, we evaluated the effects of ALA on cytokine production in the psoriatic substitutes, using a cytokine array assay that detects the levels of 36 different cytokines (Figure 5a,b). A great number of cytokines were detected in PS and PS^+T^ and the most substantial changes observed in the cytokine profile were seen in the C-X-C chemokine ligand and interleukin families, where higher levels were detected in PS^+T^. Among these, MCP-1, MIP-α, CCL5, CXCL1, CXCL10, CXCL12, ICAM-1 and IL-6 were all significantly increased in PS^+T^ when compared with PS. Conversely, the supplementation of the culture media with ALA in PS^+T+ALA^ significantly reduced the production of MCP-1, CXCL1, CXCL10 and ICAM-1, as well as the production of IL-8 and IL-6 (Figure 5a,b). The cytokines detected by the array were predominantly those produced by keratinocytes, while T cell-derived cytokines were detected in limited quantities, which is consistent with the high ratio of keratinocytes to T cells in the skin substitutes. Since cytokines can have high biological activity even at small concentrations, the levels of TNF-α and IFN-γ, two signature cytokines found in psoriasis, were subsequently measured in the supernatant of HS, PS, PS^+T^ and PS^+T+ALA^, using independent ELISA assays (Figure 5c,d) [4]. The amount of TNF-α in PS^+T^ was significantly higher than the amount in HS and PS, revealing the importance of T cells in the production of inflammatory cytokines (Figure 5c). The exogenous ALA diminished the overproduction of TNF-α in psoriatic substitutes, since significantly lower quantities of TNF-α were measured in PS^+T+ALA^ (Figure 5c). Moreover, significantly higher levels of IFN-γ were quantified in PS^+T^ as compared with PS, suggesting that IFN-γ was mainly secreted by T cells in the psoriatic model (Figure 5d). In PS^+T+ALA^, the amount of IFN-γ was less than that of PS^+T^ (Figure 5d). The levels of IL-17A, a pivotal psoriasis-related cytokine, were measured in the culture supernatants as well. Although not statistically significant, a slight decrease in the amount of IL-17A secreted was observed in PS^+T+ALA^ compared with PS^+T^ (Figure 5e). Levels of the most altered cytokines measured by the array in each skin substitutes are detailed in Table S3.

**Figure 6:**
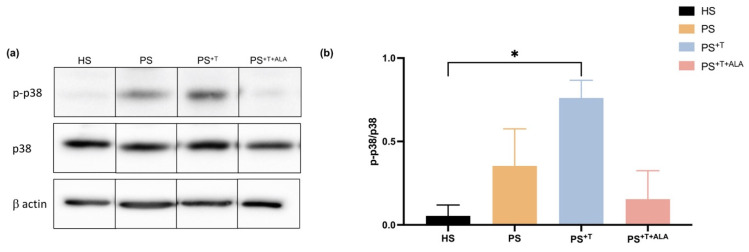
p38 protein expression in HS, PS, PS^+T^ and PS^+T+ALA^. (**a**) Twenty micrograms of total protein from skin substitutes were analyzed by immunoblot for the presence of p38 and phosphorylated p38 (p-p38). β-actin was used to control equal loading. One representative immunoblot is shown per protein. (N = 3 healthy donors and 3 psoriatic donors per condition; *n* = 2 skin substitutes per condition); (**b**) Densitometric analyses of the immunoblot from panel (**a**). Statistical significance was determined using one-way ANOVA followed by Tukey’s post hoc test. (* *p* < 0.05). Abbreviations: ALA: alpha-linolenic acid; HS: healthy substitutes; T: T cells; PS: psoriatic substitutes; PS^+T^: psoriatic substitutes produced with T cells; PS^+T+ALA^: psoriatic substitutes produced with T cells and supplemented with ALA.

To study whether ALA supplementation modulates the intracellular pathway activated following the secretion of cytokines, a specific downstream signaling pathway was investigated using Western-blot analyses (Figure 6). The levels of p38 (a protein related to the MAP kinase pathway, which is an important downstream target of CXCL1, IL-8 and IL-10 cytokines) were measured in the epidermis of the skin substitutes [41]. The ratio of phosphorylated p38 (p-p38)/p38 was increased in the PS^+T^ condition compared with its counterpart PS (ratios of 1.18 and 0.56, respectively), confirming that the presence of T cells in our model accentuated the phosphorylated state of the p38 protein (Figure 6a,b). In contrast, the supplementation of the culture media with ALA decreased the phosphorylation ratio of p-p38/p38 in PS^+T+ALA^, although not significantly. These results suggest that the effects of ALA may implicate the p38 MAP kinase pathway.

## 4. Discussion

PUFAs play valuable roles in various inflammatory diseases, and supplementation of the diet with n-3 PUFAs has been demonstrated to reduce the intensity of symptoms in psoriatic patients [13,14,16,42]. In our latest study, we have shown the effectiveness of a supplementation of the culture media with ALA in decreasing the psoriatic characteristics of our skin model produced with psoriatic fibroblasts and keratinocytes, which was represented mostly by a reduction in the biosynthesis of inflammatory lipid mediators derived from n-6 PUFAs [27]. However, the biological effects of ALA on the adaptive immunity of psoriasis, which are mainly regulated by T cells, remained to be elucidated. In the present study, we have demonstrated that ALA counteracts the hyperproliferation and abnormal cell differentiation of psoriatic keratinocytes, which were highly stimulated by T cells. Likewise, in our psoriatic skin model enriched with T cells, ALA specifically effected its beneficial effects through a decrease in the quantity of n-6 PUFAs incorporated into the phospholipid fraction of the epidermis, by diminishing the amounts of T cell-derived inflammatory cytokines, as well as by decelerating the migration of T cells into the epidermis of psoriatic substitutes.

The addition of T cells to our psoriatic substitutes highlighted the important crosstalk between keratinocytes and T cells that produces a complete inflammatory environment, mainly manifested here by hyperproliferation and an abnormal cell differentiation program, high levels of n-6 PUFAs and an increased secretion of related cytokines in PS^+T^, which are proper psoriatic characteristics [28,43]. In fact, significantly higher levels of n-6 PUFAs were measured in PS and PS^+T^ when compared with HS, as well as higher amounts of their metabolic derivative AA, which is consistent with what is found in psoriatic skin in vivo [23,44]. In addition to the high levels of n-6 lipid derivatives normally found in the cell membrane of psoriatic keratinocytes, it was reported that CD4+ T cells contain a high proportion of AA in their cell membranes, representing approximately 25% of the total fatty acids [14]. This could explain the increased n-6 PUFA levels found in PS^+T^ as compared with PS. It is known that the T cell membrane preferentially incorporates n-6 PUFAs over n-3 PUFAs during cell growth and proliferation, which correlates with our GC-FID results [45]. Furthermore, Feldon et al. reported that human T cells highly express cyclooxygenase-2 (COX-2) and can synthesize n-6 PUFA derivatives, such as prostaglandin D_2_ (PGD_2_), which further influence human skin differentiation [46]. Thus, the addition of T cells to our psoriatic skin model amplified the inflammatory properties of psoriasis by elevating the proportions of n-6 PUFAs in PS^+T^ [23]. Moreover, the expression of CD45, CD3 and ICAM-1 was detected in PS^+T^ and PS^+T+ALA^, confirming that the T cells seeded in the dermal compartment were correctly incorporated into our psoriatic substitutes. In the epidermis, T cells use various adhesion molecules, including the LFA-1/ICAM-1 interactions, to fully incorporate into the tissue [47]. In fact, the interactions between ICAM-1 on keratinocytes, and LFA-1 on T cells, mediate the binding of activated T cells in the inflamed epidermis and it is recognized that ICAM-1 is expressed de novo in the psoriatic epidermis [48]. Additionally, pro-inflammatory cytokines such as TNF-α increase T cell infiltration into inflamed skin through the induction of ICAM-1 [47].

In the current study, we focused mainly on evaluating the anti-inflammatory effects of ALA on T cell functions specifically in the psoriatic context. Supplementation of the culture media with ALA in PS^+T+ALA^ resulted in elevated ALA, EPA and n-3 DPA levels in the phospholipid fraction of the epidermis when compared with PS and PS^+T^, which implies that the addition of T cells to our psoriatic model did not affect the metabolization of ALA. This is in agreement with the mechanism of incorporation of fatty acids into epidermal phospholipids observed in native skin [49]. It was previously shown that exogenous n-3 PUFAs become incorporated into phospholipids in the plasma membrane of CD4+ T cells at the sn-2 position, which further decreases the size and stability of lipid rafts in the plasma membrane [50]. T cell plasma membranes enriched in n-3 PUFAs display many downstream perturbations in T cell activation, in particular the inactivation of the Src family kinases, as well as of the proteins Lck and Fyn [14]. The addition of ALA also reduced the quantities of AA in the epidermal phospholipids, giving support to rodent feeding studies showing that the incorporation of EPA and DHA into rodent T cell membranes reduced the AA content in a dose-dependent manner [21,51]. Therefore, since this evidence indicates that dietary n-3 PUFAs attenuate inflammation by modifying plasma membrane lipid dynamics in CD4+ T cells, the incorporation of ALA into the T cell membranes in PS^+T+ALA^ could potentially be an additional mechanism by which ALA mitigates the psoriatic features in vitro. Additionally, ALA supplementation reduced the total number of T cells in the psoriatic substitutes, as well as the migration of T cells into the epidermis, since CD45 was not detected in the epidermal fraction of PS^+T+ALA^. These results are in line with findings showing that the oral administration of n-3 PUFAs in rodents suppresses Langerhans cell migration, which further reduces the proliferation and activation of T cells in healthy skin [21,52]. Additionally, CD3-labeled T cells were present only in the dermal compartment of PS^+T+ALA^, confirming that the addition of ALA to the culture medium decreased the migration of T cells towards the dermo-epidermal junction. A recent study revealed that the percentage of CD45+ cells among CD8+ T cells was decreased in FAT-1 mice, which are capable of producing n-3 PUFAs independently [53]. The decreased expression of ICAM-1 in PS^+T+ALA^ also confirms that ALA minimizes the ability of T cells to bind to keratinocytes in the psoriatic substitutes. While the effects of n-3 PUFAs on neutrophil migration in the skin are well documented in the literature, their influence on T cell mobility has been very little studied. In fact, in 1996, De Caterina and Libby were among the first to report that the incubation of saphenous endothelial cells with 20 to 60 μM of DHA reduced the expression of ICAM-1 and VCAM-1 in a dose-dependent manner [54]. More recently, it has been determined that ALA downregulates ICAM-1 expression in both monocytes and adipose-derived stem cells, which further inhibits the secretion of IL-17A [55]. Thus, in our study, ALA seemed to reduce the number of T cells in the psoriatic substitutes by minimizing the capacity of T cells to move towards the epidermis, thereby possibly decreasing the extent of the inflammatory cascade engendered by the interactions between keratinocytes and T cells.

Globally, higher quantities of cytokines were produced in PS^+T^ compared with PS, namely of CCL5, CCL2, CXCL1, IL-8, TNF-α and IFN-γ, as reported in one of our latest studies [28]. Therefore, the addition of T cells to our psoriatic model intensifies the production of cytokines and chemokines which are synthesized by keratinocytes following their activation by T cells, generating an overall inflammatory environment more completely representative of native psoriatic skin [37,56]. We have shown that ALA influences the secretion of cytokines in our psoriatic skin model, since the added ALA significantly diminished the production of CXCL1, CXCL10, IL-6 and IL-8 as compared with PS^+T^, which is consistent with the reduced number of T cells infiltrated in PS^+T+ALA^. These results suggest that the presence of n-3 PUFAs in the culture media prevents T cells from fully producing their characteristic inflammatory mediators. In psoriatic skin, CXCL1 and IL-8 have similar roles and are recognized for activating diverse signaling pathways, such as the MAP kinase pathway, as well as for being potent chemoattractants for monocytes, dendritic cells, and T cells [16]. As for IL-6, it acts mainly on immune cell differentiation, and it is known to strongly stimulate the proliferation of CD4 helper T cells [57]. Therefore, the decreased production of these cytokines in PS^+T+ALA^ goes hand in hand with our results showing that ALA alters the infiltration and migration of T cells in our psoriatic model. It is noteworthy that keratinocytes themselves produce cytokines and chemokines, which influence the fate of T cells, and we previously reported that ALA directly disturbs the inflammatory power of psoriatic keratinocytes, namely by reducing their capacity to secrete cytokines and mediators [27]. In humans, it is recognized that a diet rich in n-3 PUFAs can suppress the production of inflammatory cytokines secreted by helper T cells as measured in whole blood, particularly that of IL-1, IL-17A, TNF-α, IL-6 and IL-2 [13,18,58,59]. In patients with periodontitis, the supplementation of the diet with 3g of EPA and 2g of DHA markedly decreased the levels of CXCL1 and CXCL10 measured in the saliva as compared with controls [60,61]. Interestingly, the levels of some cytokines such as IL-1ra, Serpin-E1 and G-CSF seemed to be decreased in models produced with T cells (PS^+T^ and PS^+T+ALA^). In fact, the role of these cytokines in psoriasis is not well defined and Serpin-E1 is associated with a Th2 T-cell signaling [62]. We believe that, in our model, T cells can deregulate the keratinocytes by increasing proliferation while decreasing differentiation, which can affect the production of some cytokines. In fact, the levels of IL-1ra increase upon keratinocyte differentiation, so the T cells added to our model could affect its production [63]. As for T-cell-specific cytokines, IFN-γ was rarely detected in PS since it is a moderator of cell-mediated immunity and therefore a powerful activator of keratinocyte mostly in the presence of T cells [4]. This result is consistent with one of our studies demonstrating that the psoriatic keratinocytes used in our model are unable to produce IFN-γ independently [36]. Herein, we have shown that ALA supplementation decreased the production of TNF-α and IFN-γ in PS^+T+ALA^, reinforcing the results obtained by Chehimi et al. [55]. In human PBMCs, an increased intake of dietary ALA inhibited TNF-α production, and it was shown that pro-resolving mediators derived from n-3 PUFAs reduce the secretion of IFN-γ [64,65]. Since lipid mediators from n-3 PUFAs regulate the secretion of cytokines, ALA potentially reduced the production of cytokines in our study by blocking the activation of the PGE_2_-EP2/EP4 signaling pathway, particularly since a decrease in AA was measured in PS^+T+ALA^ [27,66].

Furthermore, following their production, cytokines and chemokines influence a variety of biological processes. In fact, once secreted, CXCL1 and IL-8 bind to their receptor CXCR2, which results in the activation of several downstream signaling pathways, including the p38 mitogen-activated protein kinases [67]. The activation of p38 is associated with cell proliferation, and increased levels of the phosphorylated form of p38 (p-p38) are often associated with inflammation [68]. The expression of the latter was also found to be increased in native psoriatic skin [41]. In skin immunity, p38 plays a role in T cell polarization, mainly in the differentiation of Th1/Th2 cell subsets, and the persistent activation of the phosphorylated state of p38 results in increased IFN-γ production [69]. Our results are closely associated with these studies, since high levels of p-p38 were measured in PS^+T^ compared with HS and PS. Interestingly, the ratio of p-p38/p38 was decreased in PS^+T+ALA^, suggesting that the effects of n-3 PUFAs on cytokine production might extend to their underlying signaling pathways. Additionally, in prostate cancer cells, n-3 PUFAs inhibit the phosphorylation of p38, while in the skin, EPA alters p38 activation, thus demonstrating an anti-aging potential [70,71].

## 5. Conclusions

Taken altogether, our results show that ALA favorably modulated the proliferation and atypical cell differentiation of psoriatic keratinocytes, even in an environment strongly stimulated by T cells, thus emphasizing the potential of n-3 PUFAs to ameliorate the psoriatic phenotype. The added ALA was incorporated into the phospholipid fraction of the epidermis and possibly into the plasma membrane of T cells, contributing to the reduction of the inflammatory nature of psoriatic skin substitutes produced with T cells. Our study also illustrated the capacity of ALA to affect the incorporation and migration of T cells in the psoriatic model, which was represented by a decrease in the expression of CD3 and ICAM-1 in PS^+T+ALA^. Finally, we revealed that ALA regulates the secretory capacity of T cells by reducing the secretion of pro-inflammatory cytokines by both T cells and psoriatic keratinocytes. Further investigation of the modulation of signaling pathways after ALA supplementation, among others the p38-MAP kinase pathway, will be needed in order to fully detail the actions of ALA on cytokine levels. The fact remains that the use of n-3 PUFA supplements could potentially be a milder alternative to the use of certain aggressive clinical treatments such as IL-17 inhibitors, since ALA seems to modulate the secretion of this cytokine. In fact, some biological treatments, including those targeting IL-17, routinely cause side effects such as dizziness, fainting, chest tightness and fever [72]. Thus, the use of ALA supplementation could generate therapeutic effects without however causing adverse events.

## Data Availability

The data presented in this study are available directly in the article.

## Supplementary Materials

The following supporting information can be downloaded at: https://www.mdpi.com/article/10.3390/cells11091513/s1, Figure S1: K17 expression in HS, PS, PS^+T^ PS^+T+ALA^ (**a**) Twenty micrograms of total protein from skin substitutes were analyzed by immunoblot for the presence of K17. β-actin was used to control equal loading. One representative immunoblot is shown per protein. (N = 3 healthy donors and 3 psoriatic donors per condition; n = 2 skin substitutes per condition); (**b**) Densitometric analyses of the immunoblot from panel (**a**). Statistical significance was determined using one-way ANOVA followed by Tukey’s post hoc test. (* *p*-value < 0.05; ** *p*-value < 0.001; *** *p*-value < 0.0001); Figure S2: Negative controls of secondary antibodies used for immunofluorescence staining on normal human skin. Indirect immunofluorescence staining was carried out on normal human skin. Nuclei were counterstained with DAPI reagent (blue); Figure S3: CD45 expression in PS^+T^ and PS^+T+ALA^ Twenty micrograms of total protein from skin substitutes were analyzed by immunoblot for the presence of CD45. GAPDH was used to control equal loading. One representative immunoblot is shown; Table S1: Complete list of antibodies used for indirect immunofluorescence and Western-blot analyses; Table S2: Levels of fatty acids in phospholipids of skin substitutes after ALA supplementation; Table S3: Levels of most altered cytokines measured by the array in each skin substitutes. Data are represented as means ± SD.
