# Supplementary material for: Alpha-Linolenic Acid Modulates T Cell Incorporation in a 3D Tissue-Engineered Psoriatic Skin Model
Source: Cells. 2022 Apr 30;11(9):1513. doi: 10.3390/cells11091513 (PMC9104007; doi:10.3390/cells11091513)
Supplement: Supplementary file 1 [file cells-11-01513-s001.zip › cells-1703497-supplementary.pdf]

Supplementary Material

Supplementary Figures

Western blot analyses were conducted to investigate the levels of a key epidermal marker in psoriasis, namely keratin 17. Keratin 17 (K17) is an intermediate protein found in healthy basal keratinocytes and its expression spreads aberrantly into the supra-basal psoriatic keratinocytes [1]. K17 was highly expressed in PS and PS^{+T} when compared with HS. This deregulation in the level of expression of K17 is representative of what is found in psoriatic skin in vivo [1]. Our results show that ALA supplementation restored the differentiation program of psoriatic keratinocytes, since the expression of K17 was significantly downregulated in PS^{+T+ALA} compared with PS^{+T} and PS, and was closer to the level of expression found in HS.

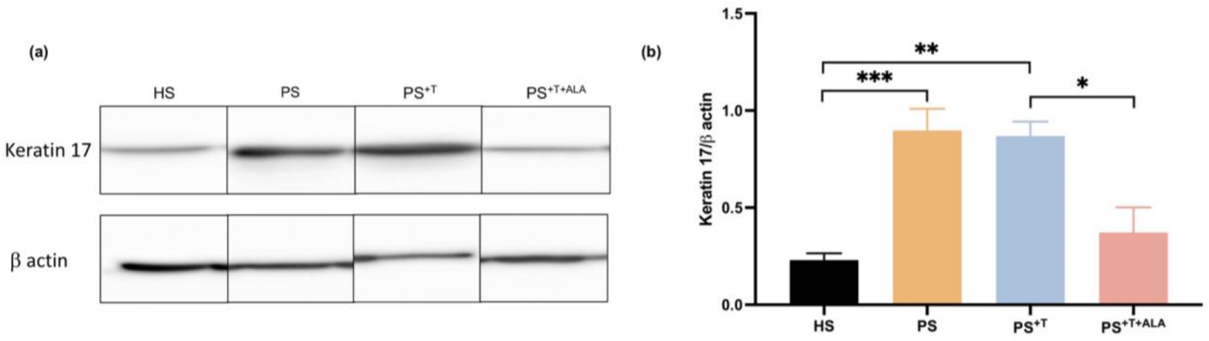

Figure S1. K17 expression in HS, PS, PS^{+T} PS^{+T+ALA} (a) Twenty micrograms of total protein from skin substitutes were analyzed by immunoblot for the presence of K17. β-actin was used to control equal loading. One representative immunoblot is shown per protein. (N = 3 healthy donors and 3 psoriatic donors per condition; *n* = 2 skin substitutes per condition); **(b)** Densitometric analyses of the immunoblot from panel (a). Statistical significance was determined using one-way ANOVA followed by Tukey's post-hoc test. (* p-value < 0.05; ** p-value < 0.001; *** p-value < 0.0001).

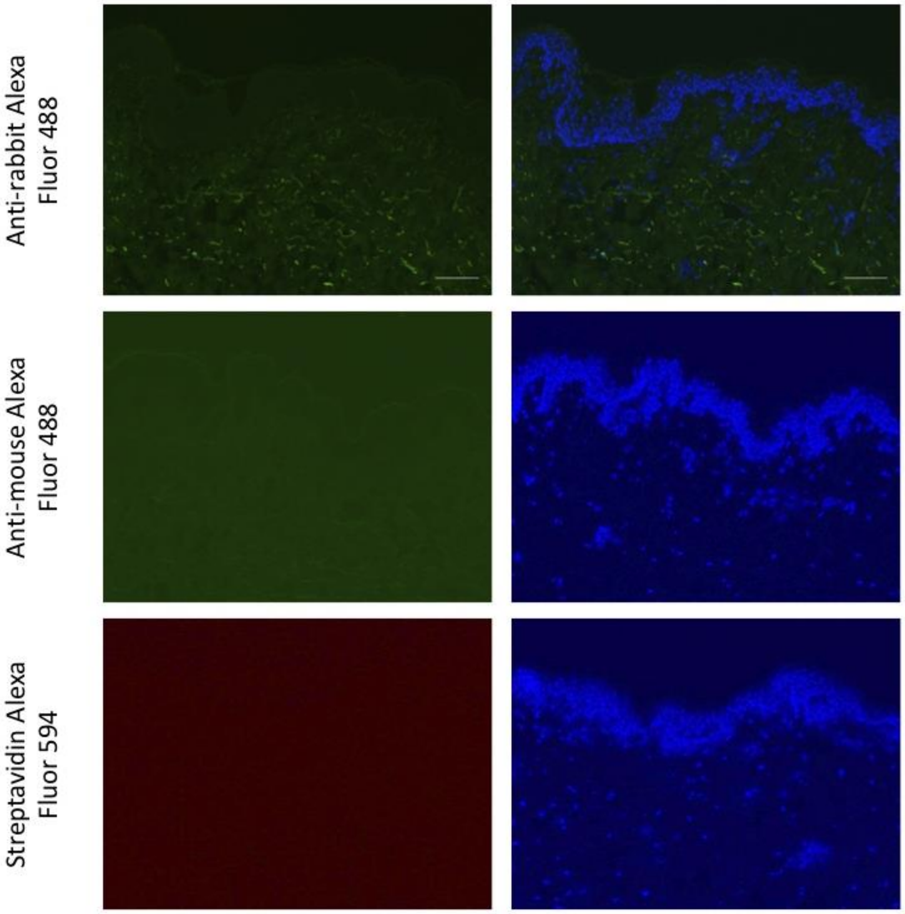

Figure S2. Negative controls of secondary antibodies used for immunofluorescence staining on normal human skin. Indirect immunofluorescence staining was carried out on normal human skin. Nuclei were counterstained with DAPI reagent (blue).

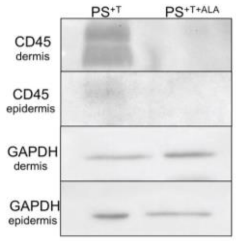

Figure S3. CD45 expression in PS^{+T} and PS^{+T+ALA} .Twenty micrograms of total protein from skin substitutes were analyzed by immunoblot for the presence of CD45. GAPDH was used to control equal loading. One representative immunoblot is shown.

Supplementary Tables

Table S1. Complete list of antibodies used for indirect immunofluorescence and western-blot analyses

Antigens	Source	Company	Catalog Number	Lot Number	Dilution
Primary Antibodies					
**Anti-human Ki67**	Mouse monoclonal IgG1	BD Biosciences	556003	8072956	1/400
**Anti-human loricrin**	Rabbit polyclonal IgG	Biolegend	905104	B243235	1/1000
**Anti-human transglutaminase 1**	Rabbit polyclonal IgG	ProteinTech	12912-3-AP	00001863	1/300
**Anti-human involucrin**	Mouse monoclonal IgG1	Sigma	I9018	103M4788	1/400
**Anti-human filaggrin**	Rabbit polyclonal IgG	Biolegend	905804	B2443692	1/500
**Anti-human CD3**	Mouse monoclonal IgG1,κ	Biolegend	344820	B240446	1/100
**Anti-human CD45**	Rabbit polyclonal IgG	Abcam	ab10558	GR155209-2	1/500
**Anti-human ICAM-1/CD54 antibody**	Rabbit polyclonal IgG	Cell signaling technology	67836	1	1/500
**p-p38**	Rabbit phosphorylated p-38 MAPK	Cell signaling technology	9211	24	1/1000
**p38**	Rabbit p38 MAPK	Cell signaling technology	9212	26	1/1000
**Anti-actin beta**	Mouse monoclonal actin beta	Sigma	A5441	079M4799V	1/30 000 (epidermis)

					and 1/10 000 (dermis)
Secondary antibodies					
**Anti-rabbit Alexa Fluor 488**	Donkey polyclonal IgG	Life Technologies	A21206	1754421	1/1600
**Anti-mouse Alexa Fluor 488**	Goat polyclonal IgG	Life Technologies	A11001	1890503	1/1600
**Streptavidin Alexa Fluor 594**	Conjugate	Life Technologies	S-11227	65B2-1	1/1000
**Anti-rabbit HRP**	Goat polyclonal IgG	Jackson ImmunoResearch Laboratories	111-035-003	-	1/60 000

Table S2. Levels of fatty acids in phospholipids of skin substitutes after ALA supplementation

**Phospholipids**					
**Mean ±SD (µg/g of Tissue)**					
**Fatty Acids**	**HS**	**PS**	**PS^{+T}**	**PS^{+T+ALA}**	***P*-Value**
**Saturated FAs**					
14:0 (Myristic acid)	36.2 ± 10.8	89.8 ± 22.4	77.4 ± 41.6	39.2 ± 24.4	NS
16:0 (Palmitic acid)	295.1 ± 117.9	639.1 ± 246.8	494.6 ± 301.4	305.4 ± 68.2	NS
18:0 (Stearic acid)	233.7 ± 89.4	453.4 ± 141.4	365.1 ± 168.9	310.4 ± 72.7	NS
20:0	10.5 ± 6.9	15.4 ± 4.3	11.6 ± 4.2	11.5 ± 4.3	NS
22:0	12.0 ± 2.8	29.3 ± 5.8	24.0 ± 7.3	22.5 ± 9.6	NS
24:0	36.2 ± 10.3	44.8 ± 35.8	39.7 ± 18.6	46.0 ± 24.8	NS
**n-3 PUFAs**					
t-18:3n-3	ND	ND	ND	ND	NS
18:3n-3 (ALA)	ND	ND	ND	23.3 ± 17.2	<0.05
20:3n-3 (ETE)	ND	ND	ND	2.845 ± 5.7	NS
20:4n-3 (ETA)	ND	0.42 ± 1.0	4.3 ± 8.5	13.2 ± 19.7	NS
20:5n-3 (EPA)	2.5 ± 2.8	5.2 ± 3.0	2.0 ± 2.7	51.6 ± 21.6	<0.0001
22:3n-3	ND	ND	ND	ND	NS
22:5n-3 (DPA)	9.1 ± 7.2	20.2 ± 6.8	28.4 ± 22.2	68.4 ± 30.0	<0.0001
22:6n-3 (DHA)	10.3 ± 7.7	20.7 ± 13.2	13.6 ± 7.1	24.4 ± 7.0	NS
**n-5 PUFAs**					
t-14:1n-5	3.1 ± 2.4	ND	ND	ND	NS
14:1n-5	ND	ND	ND	ND	NS
18:1n-5	5.2 ± 2.9	21.4 ± 4.3	24.3 ± 8.1	10.1 ± 9.3	NS
**n-6 PUFAs**					
18:1	ND	ND	ND	ND	NS
t-18:2n-6	2.8 ± 3.5	2.2 ± 3.8	3.1 ± 3.2	2.7 ± 2.7	NS
9c12t-18:2n-6	ND	7.9 ± 3.5	10.1 ± 5.3	7.5 ± 6.7	NS
8t12c-18:2n-6	4.6 ± 4.3	10.1 ± 10.6	ND	ND	NS
18:2n-6 (LA)	116.3 ± 74.0	147.5 ± 57.7	114.7 ± 62.3	173.1 ± 95.9	NS
18:3n-6 (γ-linolenic acid)	ND	4.8 ± 6.6	6.3 ± 6.6	3.7 ± 7.5	NS
20:2n-6	ND	ND	ND	ND	NS
20:3n-6 (dihomo-γ-linolenic acid)	15.2 ± 11.9	68.5 ± 19.1	65.0 ± 47.2	49.2 ± 36.4	NS
20:4n-6 (AA)	50.8 ± 41.6	160.8 ± 50.6	202.8 ± 121.6	98.7 ± 62.3	<0.001
22:2n-6	ND	ND	ND	ND	NS

22:4n-6	6.8 ± 5.4	50.1 ± 24.6	41.1 ± 14.5	18.6 ± 5.6	NS
22:5n-6	ND	11.8 ± 6.3	8.8 ± 2.9	2.8 ± 5.7	NS
**n-7 MUFAs**					
t-16:1n-7	1.0 ± 2.5	ND	ND	ND	NS
16:1n-7 (Palmitoleic acid)	177.28± 57.9	359.3 ± 76.0	346.8 ± 176.5	170.4 ± 135.5	NS
t-18:1n-7	ND	ND	ND	ND	NS
18:1n-7 (Vaccenic acid)	280.3 ± 92.6	545.1 ± 109.6	487.1 ± 201.6	247.3 ± 180.6	NS
**n-9 MUFA**					
t-18.1n-9	ND	ND	ND	ND	NS
18:1n-9 (Oleic acid)	656.1 ± 265.1	1114.3 ± 307.0	882.1 ± 409.5	560.5 ± 182.1	NS
20:1n-9	11.5 ± 4.6	19.2 ± 7.6	13.9 ± 7.6	5.3 ± 4.7	NS
22:1n-9	3.6 ± 4.0	1.0 ± 1.7	0.7 ± 1.3	ND	NS
24:1n-9	18.7 ± 6.5	35.7 ± 12.1	28.0 ± 13.0	18.0 ± 5.1	NS
**n-12 MUFA**					
t-18:1n-12	ND	ND	ND	ND	NS
18:1n-12	7.2 ± 7.6	ND	ND	ND	NS
20:1n-12	ND	ND	ND	ND	NS
**Totals**					
Total SFAs	623.6 ± 228.1	1294.2 ± 447.9	1035.2 ± 543.7	757.8 ± 142.0	NS
Total PUFAs	246.1 ± 113.3	537.8 ± 178.1	362.7 ± 148.3	389.5 ± 88.4	NS
Total MUFAs	1164.5 ± 428.6	2173.1 ± 517.8	1848.1 ± 853.6	1043.5 ± 533.9	NS
Total FAs	1913.7 ± 1021	2749.4 ± 657.9	2257.4 ± 1009	1463.7 ± 532.4	NS

Table S3. Levels of most altered cytokines measured by the array in each skin substitutes. Data are represented as means ±SD.

Cytokine	PS	PS^{+T}	PS^{+T+ALA}	*P*-value
CCL2/MCP-1	47626.12 ± 1741.2	64411 ± 738.21	46456 ± 1616.4	<0.0001
MIP-1alpha	734 ± 94.7	8367.5 ± 333.1	6677.5 ± 153.4	0.0002
CCL5/RANTES	694 ± 59.4	8427 ± 113.1	6316.5 ± 82.7	0.0001
CXCL1	31575.5 ± 2109.3	39820.5 ± 822.4	26549 ± 537.4	<0.0001
CXCL10	524 ± 267.3	42640 ± 997.0	32095.5 ± 2263.4	<0.0001
CXCL12	2230 ± 104.5	9240.5 ± 210	9104.5 ± 219.9	0.0005
ICAM-1	758.5 ± 19.1	13021 ± 130.1	8589.5 ± 188.8	<0.0001
IL-6	49421.8 ± 2038.8	53923 ± 230.5	40635.5 ± 58.7	<0.0001
IL-8	49314 ± 1204.1	43160 ± 5782.7	36332 ± 3477.6	<0.0001

Reference

1. Lin, Y.; Zhang, W.; Li, B.; Wang, G. Keratin 17 in psoriasis: Current understanding and future perspectives. *Semin Cell Dev. Biol.* **2021**, 10.1016/j.semcdb.2021.06.018, doi:10.1016/j.semcdb.2021.06.018.
